# Density dependent composition of InAs quantum dots extracted from grazing incidence x-ray diffraction measurements

**DOI:** 10.1038/srep15732

**Published:** 2015-10-28

**Authors:** Manjula Sharma, Milan K. Sanyal, Ian Farrer, David A. Ritchie, Arka B. Dey, Arpan Bhattacharyya, Oliver H. Seeck, Joanna Skiba-Szymanska, Martin Felle, Anthony J. Bennett, Andrew J. Shields

**Affiliations:** 1 Saha Institute of Nuclear Physics, 1/AF, Bidhannagar, 700064, Kolkata, India; 2 Cavendish Laboratory, University of Cambridge, J. J. Thomson Avenue, Cambridge, CB3 0HE, United Kingdom; 3 Deutsches Elektronen-Synchrotron, DESY, Notkestrasse 85, 22607, Hamburg, Germany; 4 Toshiba Research Europe Limited, Cambridge Research Laboratory, 208 Science Park, Milton Road, Cambridge, CB4 0GZ, United Kingdom

## Abstract

Epitaxial InAs quantum dots grown on GaAs substrate are being used in several applications ranging from quantum communications to solar cells. The growth mechanism of these dots also helps us to explore fundamental aspects of self-organized processes. Here we show that composition and strain profile of the quantum dots can be tuned by controlling in-plane density of the dots over the substrate with the help of substrate-temperature profile. The compositional profile extracted from grazing incidence x-ray measurements show substantial amount of inter-diffusion of Ga and In within the QD as a function of height in the low-density region giving rise to higher variation of lattice parameters. The QDs grown with high in-plane density show much less spread in lattice parameter giving almost flat density of In over the entire height of an average QD and much narrower photoluminescence (PL) line. The results have been verified with three different amounts of In deposition giving systematic variation of the In composition as a function of average quantum dot height and average energy of PL emission.

Since the discovery^1^,^2^ of narrow photoluminescence (PL) lines of InAs single quantum dots grown epitaxially on GaAs surfaces, an enormous amount of work has been carried out on these nanostructures for the development of optoelectronic devices such as lasers, quantum dot infrared photo-detectors and single-photon sources^3^,^4^. Recently, successful fabrication and operation of InAs/GaAs QD based intermediate band solar cell devices has been reported^5^,^6^. The prime challenge in this field is to determine the growth parameters to obtain predictable composition and strain profiles within a quantum dot (QD) and size-distribution of QD so that optoelectronic properties of these quantum structures can be tuned with fundamental physics calculations^7^. It has been pointed out recently that the In-Ga intermixing within the self-assembled QD strongly influences intensity distribution and polarization of emitted photons^8^ as the confinement length of the carriers depend mainly upon the In composition profile within a QD and not just on their size^9^.

Several structural studies have already established that InAs QD grow on GaAs substrate in Stranski-Krastonov (SK) growth mode by forming InGaAs wetting layer (WL), initially, at lower growth temperatures (around 350 °C)^10^. It is also known that at higher growth temperatures (above 420 °C), the QD volume becomes much higher than the deposited additional InAs after formation of WL, providing a direct evidence of considerable migration and intermixing of In and Ga^11^,^12^. Substantial intermixing of In and Ga was also observed in thickening of WL after QD formation^13^. High resolution transmission electron microscopy measurements^14^ and scanning tunneling electron microscopy studies^15^,^16^ have indicated segregation of In towards the center and tip of the QD formed above the WL. It is essential now to understand role of various growth conditions, like the deposition rate, temperature and in-plane density to tune the size and composition of these QD for obtaining desired electronic, structural and optical properties^7^,^17^.

In-plane density of self-assembled InAs QDs on GaAs surface is an important parameter for the development of devices based on these nanomaterials. Dense and uniform array of QD may be useful for solar-cells or lasers but low density limit may be valuable in the development of devices for quantum information processing. Recently it has been demonstrated that such density dependent growth can be characterized by Hopkins-Skellan Index^18^. Here we show that composition and strain profile and also PL property of QD can be tuned by controlling the in-plane density of QD by having a growth temperature gradient over the substrate. Results of grazing incidence x-ray scattering (GIXS)^19^,^20^,^21^ measurements of three representative samples presented here show that composition and morphology across the height of an average QD are quite different in the low-density and in the high-density regions (refer Fig. 1). The GIXS measurements provide significant insight into the In-composition profile in an average QD and also provide valuable information regarding the strain profile as a function of height within an average QD. We present here results of two types of GIXS measurements (refer Fig. 2) on six different dot-densities (refer Fig. 1), namely grazing incidence diffraction (GID) to extract In composition profile as a function of in-plane lattice parameter and grazing incidence critical angle measurement to map in-plane lattice parameter to average QD heights. The GIXS results show substantial amount of inter-diffusion of Ga and In within the average QD in all the three samples as a function of dot heights.

All the presented three samples w0795, w0808 and w0809 have multilayer structure with two layers of InAs quantum dots separated by GaAs buffer layers deposited on GaAs (001) substrate. It should be mentioned here that the PL peak is obtained here from the buried lower layer of quantum dots and the exposed top layer of dots was probed by GID and AFM measurements. The details of the sample structure and growth conditions are given in the ‘Methods’. Due to heat sinking effects resulting in a temperature gradient^22^ it was found that the edge of the substrate was 15 °C colder than the centre. Moreover an intentional non-uniformity in the Indium source flux was used to supply around 10% less In at the edge of the substrate. The difference between the three samples was only the In deposition time, which was kept equal for both the buried and exposed QD layers. For the presented samples w0795, w0808 and w0809 the In deposition time were 115, 105 and 100 seconds respectively. The GID measurements provide significant insight into the In-composition profile in an average QD and also provide valuable information regarding the strain profile as a function of height within an average QD for the three samples. PL measurement results presented here provided us information regarding optical properties of the buried QD layer as a function of in-plane QD density. The conclusions drawn here assume that both the buried and exposed layer of quantum dots have similar structural and compositional profiles. In future, we plan to study GID, AFM and PL from same QD layer by reducing the thickness of the cap-layer to improve our understanding.

## Results and Discussion

**Figure 1:**
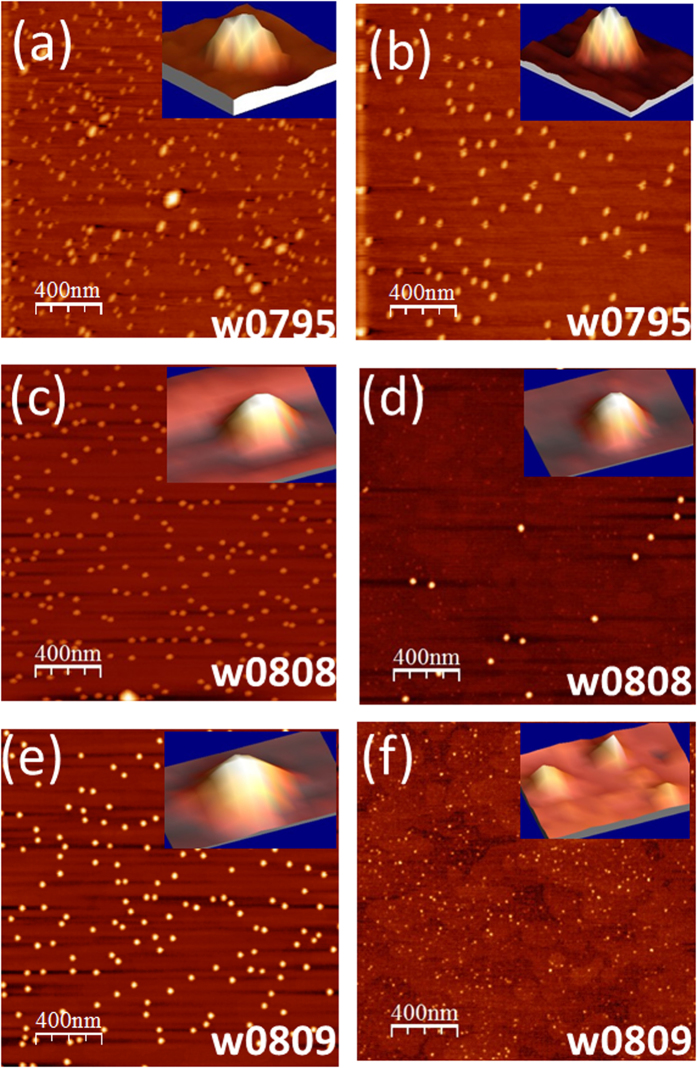
2 *μ*m × 2 *μ*m AFM images taken from the edge (left panel) and centre (right panel) of InAs quantum dots formed on the surface of each wafer used in the study. (**a**,**b**) W0795 (115 s deposition), (**c**,**d**) W0808 (105 s), (**e**,**f**) W0809 (100 s). Insets show the 3-D view of a single QD in the respective low and high QD density regions.

We first present the results of AFM measurements of the three InAs QD samples having different coverage at the edge and at the center positions in Fig. 1. The left and right panels correspond to the edge and central regions respectively on the substrate surface exhibiting different in-plane densities. The QD at both positions are found to be elongated in shape for w0795. High density QD obtained at the edge of the substrate surface are observed to have diameters roughly 31 ± 2 nm and 50 ± 2 nm in the two mutually perpendicular directions and the average height was found to be 17 ± 4 nm. Presence of very few dome sized islands could also be observed. Low density QD obtained near the center of the substrate with higher growth temperature have diameters of around 38 ± 2 nm and 60 ± 3 nm in the two mutually perpendicular directions with average height of 25 ± 2 nm. The QD in this region are seen to be more uniform in size and the dome sized islands are completely absent here. This can be understood by the fact that at higher temperature, the adatoms have higher diffusivity leading to the coalescence of small islands to form larger QD having uniform size^22^,^23^. The higher mobility of the adatoms on the central region of substrate surface also leads to a lowering in-plane density of the QD from 100 per μm^2^ at the edge to 50 per μm^2^ at the center. The central and edge portions of the substrate surface will now be referred as low-density and high-density regions, respectively. Relatively spherical quantum dots are observed in the sample w0808. Low density QD observed at the centre of the substrate consist of larger QD having average diameter of 56 ± 1 nm and height of 18 ± 1 nm. This central region also consists of wet layer pre-pyramids which are observed to be around 2 nm high with diameter of around 26 ± 2 nm. At the substrate edge, the average QD diameter is observed to be 53 ± 2 nm with a height of 15 ± 1 nm. The in-plane number density of QD is observed to be 5 per μm^2^and 30 per μm^2^at the central and edge regions respectively of the sample. For the sample w0809, having the lowest In deposition among all, wet layer with several pre-pyramids (having average height of around 2 nm) can be observed at the central portion of the substrate. The edge region of the substrate is observed to have a very high in-plane QD number density of 42 per μm^2^. Again, slightly elongated QD are observed with average diameters of 68 ± 1 nm and 56 ± 1 nm in the two mutually perpendicular directions and average height of 16 nm. Insets of Fig. 1 show the 3-D view of a representative single QD in the high and low density regions, respectively for all the three samples.

**Figure 2:**
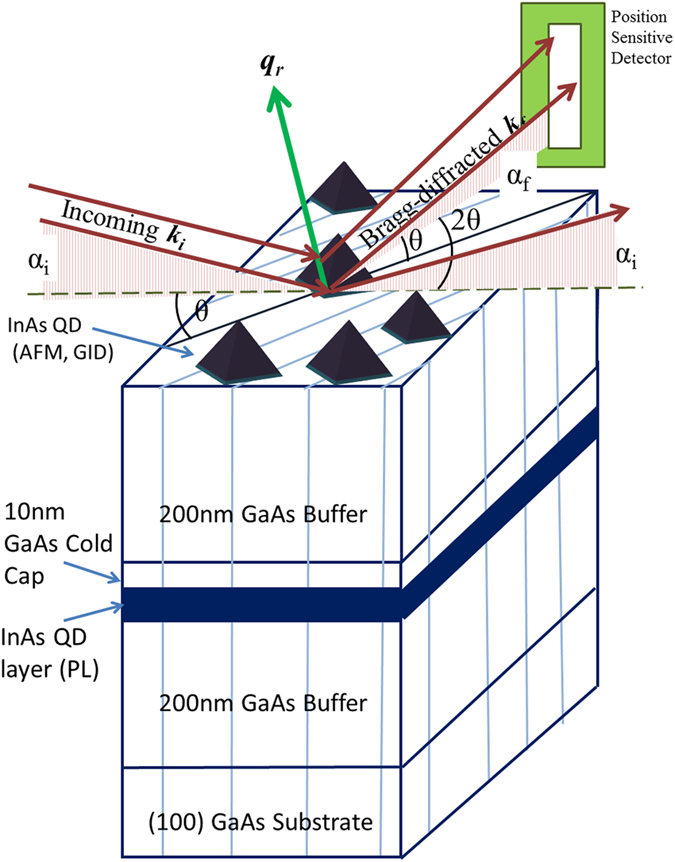
Schematic of the grazing incidence diffraction measurements on the quantum dots present on the top surface of the structure. The details of the sample structure has also been indicated (refer text for details).

**Figure 3:**
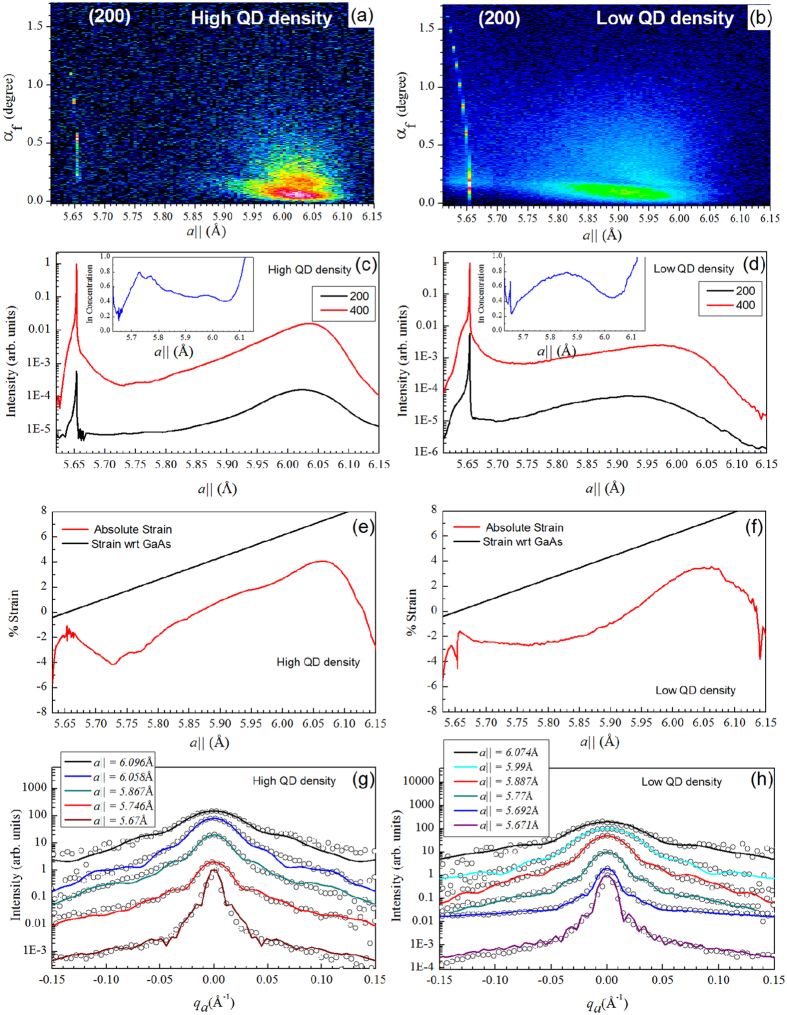
(**a**,**b**) show two-dimensional GID data around (200) for the high and low QD density regions respectively. Y-axis represents the exit angle in the Mythen detector. (**c**,**d**) Line profile of the radial scans around (200) and (400) as indicated for the high and low QD density regions respectively. These line profiles are generated by the integration of all the channels of Mythen detector. Respective insets show the variation of In concentration with *a*_*||*_. (**e**,**f**) Profiles of the absolute strain present in the QD and strain with respect to GaAs (refer text for the method of calculation) as a function of *a*_*||*_ for the high and low QD density regions respectively. (**g**,**h**) Angular scans around (400) corresponding to different in-plane lattice parameters as indicated for the high and low QD density regions respectively.

The schematic diagram of the GIXS measurements and the sample having a layer of the InAs quantum dots at the top surface of the grown structure is shown in Fig. 2. Figure 3(a,b) show the representative 2-D plots of the radial scans around (200) GID peak at high and low density regions for w0795 sample, respectively. The scattered intensity variation with respect to the in-plane lattice parameter (X-axis) and the detector exit angle (α_f_) (Y-axis) are found to be quite different in these two density-regions. In the high-density region, the scattered intensity is concentrated over a small lattice parameter space (from *a*_*||*_ = 5.95 to 6.08 Å) but for low-density region, scattering was observed over large in-plane lattice parameters (*a*_*||*_ = 5.77 to 6.02 Å). Figure 3(c,d) represent typical radial scans around (400) and (200) GID peaks for the QDs grown at the high and low density regions respectively. The line profiles shown here are obtained by integrating the intensity along detector exit-angle (α_f_). The structure factors for the two reflections can be written as *F*_*400*_ = *x f*_*In*_ + (1−*x*) *f*_*Ga*_ + *f*_*As*_ and *F*_*200*_ = *x f*_*In*_ + (1−*x*) *f*_*Ga*_*—f*_*As*_ where *x* is the concentration of In present in the QD and *f*_*Ga*_, *f*_*As*_ and *f*_*In*_ are the atomic scattering factor for Ga, As and In respectively. By computing the intensity ratio (*I*_400_/*I*_200_) around the two GID peaks (400) and (200), one can extract the In content (*x*) inside the In_*x*_Ga_*1-x*_As QD as a function of in-plane lattice parameter as:


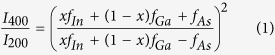


Insets in Fig. 3(c,d) show the variation of In content calculated using this expression for high and low density regions respectively. The In concentration increases to (*x* ≈ 0.8) with increasing in-plane lattice parameter and then reduces to (*x* ≈ 0.4) towards the InAs lattice parameter for both low and high density regions. However the extracted compositional profiles for two density regions are found to be quite different.

The absolute in-plane strain present in the QD can be calculated by *ε*_*||*_ = [*a*_*||*_ *−* *a*(*x*)]*/a*(*x*) where *a*_*||*_ is the in-plane lattice parameter and *a*(*x*) is the lattice parameter corresponding to the In content (*x*) given by Vegard’s law^24^. The in-plane strain-profile was also calculated with respect to the substrate (GaAs) lattice parameter given by *ε*_*||*_ = (*a*_*||*_ − *a*_*GaAs*_)*/a*_*GaAs*_. These calculated strain-profiles are presented in Fig. 3(e,f) for high and low density regions respectively. The absolute in-plane strain profiles of QD present in both density regions were found to evolve from highly compressive to tensile in nature. It can be observed from the Fig. 3(e) that the strain value varies from −4 (compressive strain) to +4 (tensile strain) with a high slope for the high density region while for low density region it varied from −2 to +3 with the increase in lattice parameter (refer Fig. 3(f)). Thus, the QDs are more relaxed in low density region than those present in the high density region. This observation is consistent with the fact that higher In-Ga intermixing occur in the low density region due to higher growth temperature.

**Figure 4:**
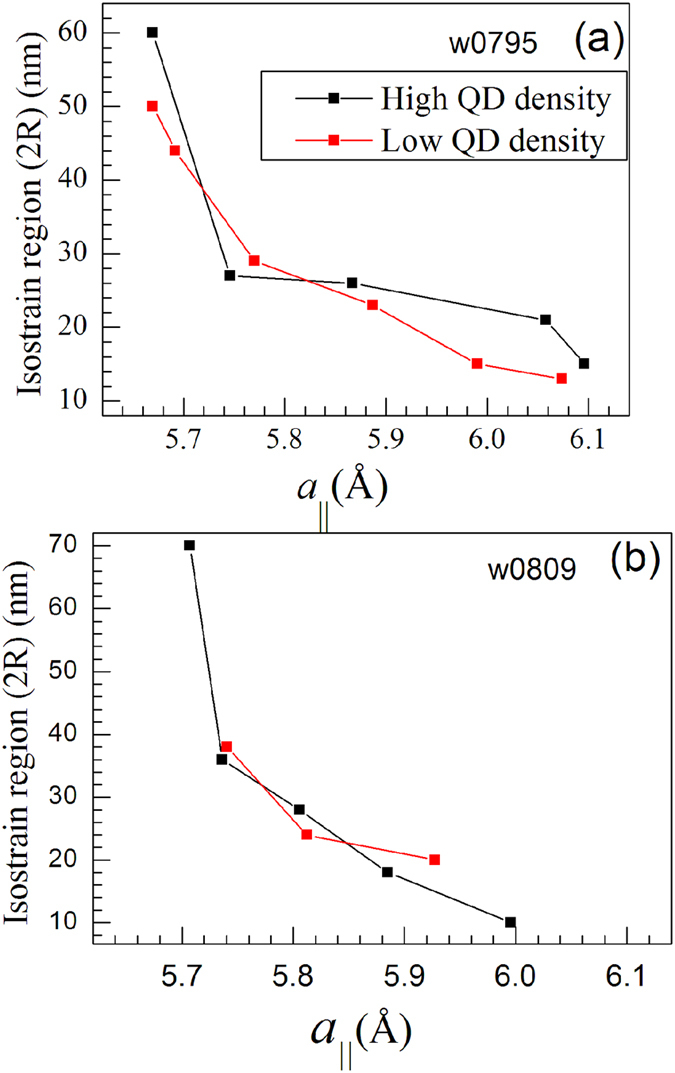
The variation of iso-strain disk diameter (2R) with *a*_*||*_ for the two density regions (**a**) sample w0795 and (**b**) sample 0809.

**Figure 5:**
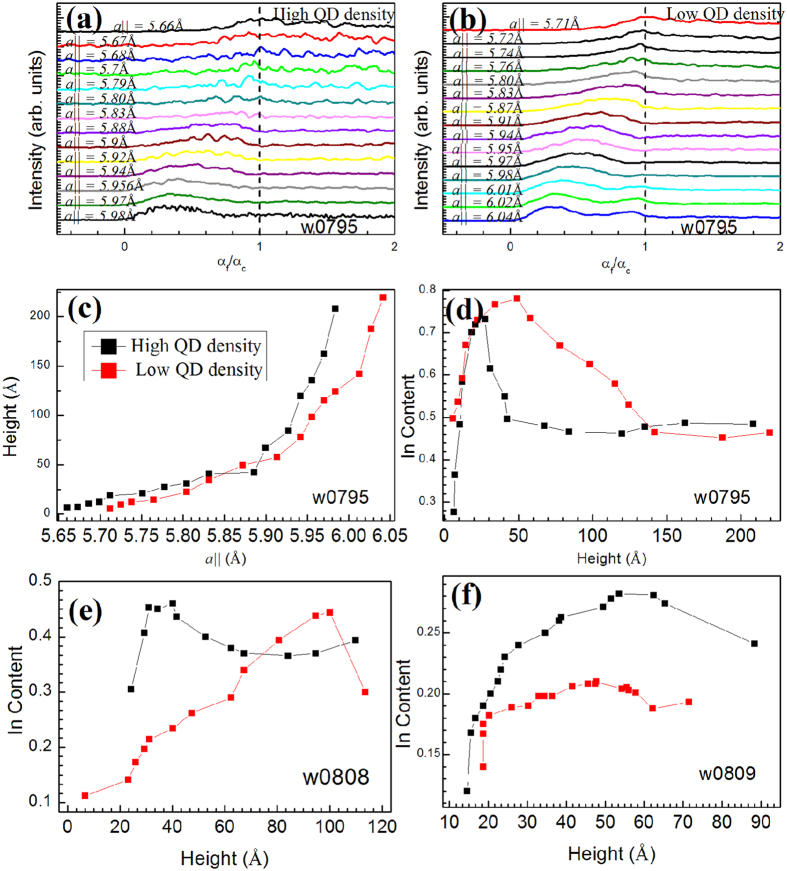
(**a**,**b**) Experimental exit angle (α_f_) intensity profiles corresponding to different *a*_*||*_ for high and low QD density regions respectively for sample w0795. (**c**) Height with respect to the GaAs substrate (refer text for calculation) for different *a*_*||*_ for sample w0795. Variation of In content with height inside the QD for the two regions for (**d**) sample w0795 (**e**) sample w0808 and (**f**) sample w0809.

Since the quantum dots under investigation are epitaxial, the base of the QD will have in-plane lattice parameter close to that of GaAs (*a*_GaAs_ = 5.653 Å) and towards the apex of QD, the lattice would tend to approach the value of InAs (*a*_InAs_ = 6.058 Å). Thus, the lattice near the base of InGaAs QD will suffer compressive strain and that near the apex of QD will experience tensile strain. It is known that for InGaAs QD, In content is lower in the apex of QD and higher in the middle region^11^,^14^. For simplicity of analysis, here we assume an average QD consists of few disks stacked one over the other, each having a unique lattice parameter^24^. Figure 3(g,h) show typical angular scans at the high and low density regions respectively around (400) GID peak at several fixed radial momentum positions corresponding to different in-plane lattice parameters (*a*_*||*_) as indicated. These intensity plots as a function of angular momentum provide us the isostrain length scales i.e. the length of the area having same in-plane lattice parameter which can be considered to be the diameter of the disks in the model. The contribution of each disk with radius ‘R’ in the obtained x-ray scattering profile can be given by^24^,^25^:


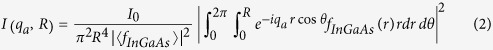


where *f*_InGaAs_(r) is the effective scattering factor at position ‘*r*’ from the center of the disk. The line profiles in Fig. 3(g,h) show the fit of the angular momentum intensity profiles as calculated using Equation (2). Similar calculations and data analysis were performed for the samples w0808 and w0809 as well and the results have been presented in Figs 4 and 5 along with results of the w0795 sample. Figure 4(a,b) show the variation of the isostrain region with *a*_*||*_ for the two density regions for samples w0795 and w0809 respectively. It can be seen that as the in-plane lattice parameter (*a*_*||*_) increases, the radii of the disks constituting an average QD decrease for both the samples. This coincides with the fact that the lower *a*_*||*_ values represent the base region of the QD and the higher *a*_*||*_ values correspond to the QD-apex. In Fig. 4(a) for sample w0795, the diameter of 50 nm for the base of QD in low density region matches quite well with the average value obtained from AFM measurements. However, the value of 60 nm for the base diameter in the high density region is higher than that estimated from the AFM measurements. It should be noted here that in the high QD density region, the islands have varied sizes and occurrence of large sized domes is also evidenced. It is also to be noted that x-ray techniques provide statistically averaged results over large areas and also probe buried layer as compare to AFM measurements that yield localized information of the exposed surface. Figure 4(b) shows the variation of isostrain region with *a*_*||*_ for the sample w0809. The highest diameter of 70 nm for the high QD density region matches well with that obtained from the AFM measurements. For the low QD density region, the maximum isostrain region is found to be around 38 nm which corresponds to the pre-pyramids observed in this region from the AFM measurements.

The position of a disk in the model, with given *a*_*||*_, above the GaAs surface can be estimated from the exit angle plots^19^,^20^ of the Mythen detector. Figure 5(a,b) show the exit angle plots [in (400) GID geometry] corresponding to different in-plane lattice parameters (as indicated) for the high and low density regions respectively for sample w0795. These exit angle intensity profiles can be extracted directly from the radial scans as their 2-D plots [refer Fig. 3(a,b)] show intensity variation with respect to α_f_ and *a*_*||*_. From the position of the first maximum (*α*_f_^max^), the height ‘z’ above GaAs surface corresponding to any *a*_*||*_ can be calculated as^19^,^20^:


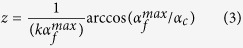


where *k* is the wave number of the x-ray beam and α_c_ is the critical angle for GaAs. Thus, the height of a particular iso-strain region in the QD from the GaAs surface can be calculated using Equation (3) and is represented in Fig. 5(c). As the in-plane lattice parameter increases, the height inside the QD also increases monotonically. Thus, from base to apex of the QD, the in-plane lattice parameter (*a*_*||*_) increases and the diameter of the QD decreases as can be inferred from Figs 4(a) and 5(c). Similar trend was observed for the w0809 sample having minimum In deposition as shown in Fig. 4(b) though over much reduced range. The diameters corresponding to the highest in-plane lattice parameter in both density regions correspond to the apex region of the QD. Also, it is observed that the apex of the QD corresponding to the *a*_*InAs*_ (= 6.05 Å) is under high tensile strain for both the QD regions [refer Fig. 3(e,f)].

**Figure 6:**
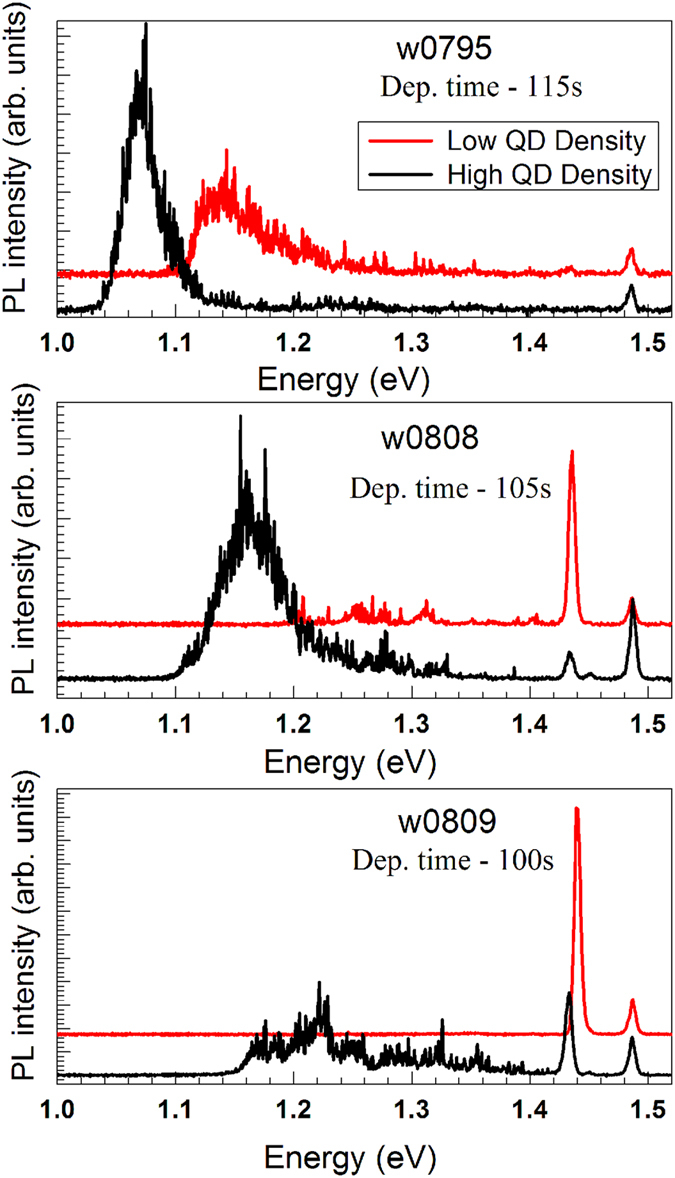
Photoluminescence spectra from the low and high QD density regions for each of the three samples. In the low density region (centre of the substrate) it is clear that the dot density is reduced significantly as the deposition thickness is reduced. In (**a**) large QDs emitting at ∼1.1 eV with no wetting layer (WL) emission are observed. In (**b**) the density is sufficiently low to observe individual dot lines around 1.16 eV with WL emission at 1.44 eV. In (**c**) there is no eVidence of QD emission only a strong WL signal is observed.

By comparing the in-plane lattice parameter with those in the insets of Fig. 3(c,d) respectively for the high and low density regions, we could correlate different angular scans [Fig. 3(g,h)] with the height dependent In-profile of an average QD. In Fig. 5(d–f) we have shown the extracted In profile within an average QD as a function of height for samples w0795, w0808 and w0809 respectively. It is observed that the In content for an average QD in both density regions increase initially as a function of height measured from the base. This observation is consistent with the previous XTEM studies and theoretical calculations performed on InGaAs QD which show a segregation of Indium in their central region^11^,^14^. For the QD grown at the edge having higher in-plane density, the In content increases from base and then fall within 5 nm to attain a nearly constant value until the apex of QD. The highest Indium content (*x* = 0.74) in the high density region was found at the height of 3 nm above the substrate and it corresponds to the iso-strain disk of radius 13 nm for sample w0795. On the other hand for low density region of this sample highest Indium content (*x* = 0.78) was obtained at the height of 8 nm above GaAs substrate corresponding to the iso-strain disk of radius 11.5 nm. Moreover these low density QDs grown in the central portion of the substrate exhibit high In content even beyond 10 nm height and finally attain a constant value (*x* = 0.4) towards the tip. Almost constant value of Indium content beyond the base height of 5 nm of average QD in high density region, apparent in Figs 3(a) and 5(d), was found to be crucial in exhibiting sharper photoluminescence (PL) shown in Fig. 6. For the samples w0808 and w0809 (refer Fig. 5(e,f) respectively), the In concentration increases initially from the base of the QD in both the low and high QD density regions and decreases towards the apex as observed for the sample w0795. Lower In deposition time resulting in lesser In content is quite evident from these results as the maximum In content within the QD for w0795 is observed to be 0.8 while the highest In content in w0808 is observed to be 0.46 and that in sample w0809 it is 0.28. A decrease in QD height is also observed as the In deposition is reduced as expected.

In Fig. 6 we have shown the micro PL spectra for all the three samples measured at the two density regions. A picoquant 785 nm laser diode driven at 80 MHz was used as the excitation source for collecting PL from a sample placed in an Oxford Instruments continuous flow cryostat. The sample temperature was 10 K. The signal was directed to a Horiba HR460 spectrometer and detected by a LN_2_ cooled InGaAs array. The PL peaks are observed to be at 1.07 eV (FWHM = 0.038 eV) and 1.14 eV (FWHM = 0.058 eV) for the high and low density regions respectively for sample w0795. The peak position is observed to be shifted to lower energy giving sharper peak for the high density region as compared to that for low density region. Following earlier results^26^,^27^, we conclude that broad PL peak observed in low density region can be attributed to the higher In-Ga interdiffusion observed in x-ray measurement (refer Fig. 4(d) of this region). Further study of crystal defects such as dislocations that may lead to the shifting of PL peak energy to higher values is required to substantiate this observation as higher InAs deposition on the central portion of the substrate surface may lead to such defect states^28^,^29^. For samples w0808 and w0809 with lower In deposition large fall in PL intensity was observed. Only WL related emission is observed in sample w0808 from low density region at around 1.44 eV. For high QD density region of this sample a broad PL intensity distribution is observed around 1.22 eV. For w0809 sample, a broad QD related peak is observed around 1.16 eV (FWHM = 0.057 eV) and a small WL emission is observed. This shifting of high QD density related PL emission to higher energies may be attributed to the lower In content in the samples w0808 and w0809 as compared to w0795.

## Conclusions

Epitaxial InAs quantum dots grown on same GaAs wafer at different deposition temperatures have been studied. AFM measurements suggest the coarsening of small quantum dots to form uniformly sized larger quantum dots for the higher deposition temperature. This coarsening and absence of it lead to variation in In-Ga intermixing inside the quantum dots deposited at different growth temperatures. Results of grazing incidence x-ray scattering measurements presented here clearly show that quantum dots grown in low-density region have large variation of Indium composition as a function of height in an average dot. Most promising results were obtained from w0975 sample where Indium concentration profile within an average quantum dot present in high in-plane density region exhibit a sharp In-peak near the base of the dot and then a flat In_0.4_Ga_0.6_As composition over the rest of the dot giving a much sharper PL emission as compare to the dots grown in low-density region. The techniques developed here to correlate GIXS and PL measurements of quantum dots will help to develop better structure-spectroscopy relationship in these technologically important materials.

## Methods

### Sample Preparation

The samples were grown by molecular beam epitaxy using a Veeco Gen III system on 3 inch semi-insulating GaAs (001) substrates. The structure consists of a 250 nm GaAs buffer grown at 580 °C, a QD layer plus 10 nm GaAs capping layer grown at 515 °C for PL measurements followed by a second 200 nm GaAs buffer and a surface layer of QD is deposited at 515 °C for AFM and GID studies. The GaAs growth rate was 1 ML/s and the InAs arrival rate was 0.027 ML/s. Due to the relatively high deposition temperature for the InAs QD resulting in some desorption, the Indium shutter was kept open for a time equivalent to 0.8625 nm (2.8 ML) for both the layers of QDs in sample w0795. Similarly for both layers of w0808 and w0809 samples shutter was open for growth of 0.7875 nm and 0.75 nm, respectively. For the sample w0809, having the lowest In deposition among all, wet layer with several precursors are observed at the central region of substrate^30^,^31^.

### X-ray measurements

Synchrotron measurements were performed at the two different QD density regions on the sample surface to study their size, strain profile and the amount of In-Ga intermixing as a function of the height of QD. All x-ray experiments were performed at Beamline P08 of Petra III synchrotron in DESY, Germany at energy of 11103 eV^32^. A beam-defining slit setting of dimension 50 × 300 micron was used in vertical and horizontal direction respectively and the data was collected by a position sensitive linear Mythen detector. The intensity of all the channels was integrated to obtain the data presented here. During measurements the incident angle was kept to be 0.1° which is lower than the critical angle for GaAs, in order to keep the x-ray beam onto the surface of the sample to enhance sensitivity to the InGaAs QDs present at the top surface. The obtained results of radial and angular scans taken in GID geometry around two in-plane diffraction peaks around (400) and (200) are presented.

Radial scans are intensity measurements in which incidence angle to the planes in the sample surface (*θ*) and detector angle (*ϕ*) are varied by keeping *ϕ* = 2*θ*. The intensity measurement by this type of scans is directly related to the in-plane lattice parameter (a_||_) defined by 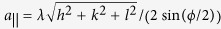, where (*h,k,l)* are the Miller indices of the nearest Bragg reflection in GID geometry. In angular scans, θ is varied by keeping 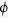 value constant and these data give the size of the region in the sample having a fixed lattice parameter corresponding to the fixed 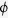 position having 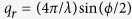. Only the angular momentum transfer 
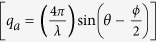
 changes in such a scan and the radial momentum remains constant.

## Additional Information

**How to cite this article**: Sharma, M. *et al.* Density dependent composition of InAs quantum dots extracted from grazing incidence x-ray diffraction measurements. *Sci. Rep.* **5**, 15732; doi: 10.1038/srep15732 (2015).
